# Dynamic changes of hepatic vein Doppler velocities predict preload responsiveness in mechanically ventilated critically ill patients

**DOI:** 10.1186/s40635-024-00631-w

**Published:** 2024-05-08

**Authors:** Mario Bruna, Sebastian Alfaro, Felipe Muñoz, Liliana Cisternas, Cecilia Gonzalez, Rodrigo Conlledo, Rodrigo Ulloa-Morrison, Marcos Huilcaman, Jaime Retamal, Ricardo Castro, Philippe Rola, Adrian Wong, Eduardo R. Argaiz, Roberto Contreras, Glenn Hernandez, Eduardo Kattan

**Affiliations:** 1 Unidad de Cuidados Intensivos, Hospital de Quilpué, Quilpué, Chile; 2 Facultad de Medicina, Escuela de Medicina, Universidad Andrés Bello, Viña del Mar, Chile; 3 Departamento de Medicina Intensiva, Facultad de Medicina, Pontificia Universidad Católica de Chile, Avenida Diagonal Paraguay 362, Santiago, Chile; 4 Unidad de Cuidados Intensivos, Hospital Las Higueras, Talcahuano, Chile; 5 Unidad de Cuidados Intensivos, Hospital Gustavo Fricke, Viña del Mar, Chile; 6 Intensive Care Unit, Hopital Santa Cabrini, CIUSSS EMTL, Montreal, Canada; 7 Department of Critical Care, King’s College Hospital, London, UK; 8 Departamento de Nefrología y Metabolismo Mineral, Instituto Nacional de Ciencias Médicas y Nutrición Salvador Zubirán, Mexico City, Mexico; 9 Tecnológico de Monterrey, Escuela de Medicina y Ciencias de la Salud, Mexico City, Mexico; 10 Unidad de Cuidados Intensivos, Hospital Biprovincial Quillota-Petorca, Quillota, Chile

**Keywords:** Passive leg raising, Hepatic vein Doppler, Preload responsiveness tests, Shock

## Abstract

**Background:**

Assessment of dynamic parameters to guide fluid administration is one of the mainstays of current resuscitation strategies. Each test has its own limitations, but passive leg raising (PLR) has emerged as one of the most versatile preload responsiveness tests. However, it requires real-time cardiac output (CO) measurement either through advanced monitoring devices, which are not routinely available, or echocardiography, which is not always feasible. Analysis of the hepatic vein Doppler waveform change, a simpler ultrasound-based assessment, during a dynamic test such as PLR could be useful in predicting preload responsiveness. The objective of this study was to assess the diagnostic accuracy of hepatic vein Doppler S and D-wave velocities during PLR as a predictor of preload responsiveness.

**Methods:**

Prospective observational study conducted in two medical–surgical ICUs in Chile. Patients in circulatory failure and connected to controlled mechanical ventilation were included from August to December 2023. A baseline ultrasound assessment of cardiac function was performed. Then, simultaneously, ultrasound measurements of hepatic vein Doppler S and D waves and cardiac output by continuous pulse contour analysis device were performed during a PLR maneuver.

**Results:**

Thirty-seven patients were analyzed. 63% of the patients were preload responsive defined by a 10% increase in CO after passive leg raising. A 20% increase in the maximum S wave velocity after PLR showed the best diagnostic accuracy with a sensitivity of 69.6% (49.1–84.4) and specificity of 92.8 (68.5–99.6) to detect preload responsiveness, with an area under curve of receiving operator characteristic (AUC–ROC) of 0.82 ± 0.07 (*p* = 0.001 vs. AUC–ROC of 0.5). D-wave velocities showed worse diagnostic accuracy.

**Conclusions:**

Hepatic vein Doppler assessment emerges as a novel complementary technique with adequate predictive capacity to identify preload responsiveness in patients in mechanical ventilation and circulatory failure. This technique could become valuable in scenarios of basic hemodynamic monitoring and when echocardiography is not feasible. Future studies should confirm these results.

**Supplementary Information:**

The online version contains supplementary material available at 10.1186/s40635-024-00631-w.

## Background

Expert recommendations suggest that resuscitation strategies should include the assessment of dynamic parameters to identify patients who will significantly increase cardiac output (CO) after fluid administration [1, 2]. Different techniques to predict preload responsiveness have been described in the literature [3] by testing reversible changes in preload and assessing its impact on CO or its surrogates. Each of these tests have variable diagnostic accuracies, technical nuances, and most important, often limited clinical contexts in which they can be adequately applied at the bedside [4].

Among them, passive leg raising (PLR) has become one of the most used preload responsiveness tests, due to its applicability in a wide array of scenarios, including patients in spontaneous ventilation, low tidal volume ventilation or presence of arrythmias [5]. Unfortunately, the maneuver requires either real-time CO [6] measurement through devices that are not routinely available in all intensive care units (ICU) [7], or ultrasound assessment of left ventricular outflow tract–velocity time integral (LVOT–VTI) [8], which is not always feasible to measure correctly [9]. This may be accentuated during PLR’s fast position change of the thorax, which makes image acquisition more challenging.

The Guytonian model of circulation defines that at a steady state, CO is equal to venous return (VR) [10, 11]. Moreover, directly assessing VR in daily clinical practice has proven elusive [11], even though VR is constantly manipulated by administering both fluids and vasopressors. Recent interest has emerged on the analysis of hepatic vein Doppler (HVD) through surface ultrasound as a novel window to assess physiological and pathological cardiovascular states [12, 13]. It has a specific Doppler envelope, where S and D waves represent anterograde blood flow to the right chambers during the cardiac cycle [14], and presents a high image acquisition rate [15]. Thus, tracking changes in HVD after a dynamic test such as PLR could effectively aid clinicians to identify significant changes in VR (and thus, CO), potentially predicting preload responsiveness. Unfortunately, there is paucity of data assessing this phenomenon.

The objective of this observational bi-centric prospective study was to assess the diagnostic accuracy of the changes of HVD S and D-wave velocities during a PLR maneuver as predictor of preload responsiveness in mechanically ventilated critically ill patients requiring hemodynamic resuscitation. We hypothesized that changes in HVD S and D-wave velocities would accurately predict preload responsiveness in this study population.

## Methods

The study protocol was approved by the Comité Ético Científico HGF‐SSVQ (N: 07/2023), and Comité Ético Asistencial UC (N°: 220923006) and written informed consent was waived due to the observational and non-invasive nature of the study design. This study was conducted in accordance with the 1964 Declaration of Helsinki and followed the STARD guidelines for diagnostic accuracy studies [16].

### Patients

Subjects older than 18 years of age who were admitted to the ICU of Hospital de Quilpué, in Quilpué, Chile and Hospital Clínico UC-Christus, in Santiago, Chile requiring controlled mechanical ventilation, in circulatory failure, and in which the attending physician performed a PLR with cardiac output assessment (PLR–CO) as part of routine clinical care were considered eligible for this study. Circulatory failure was defined as the need for vasoactive drugs to maintain MAP ≥ 65 mm Hg and the presence of at least one clinical sign of inadequate tissue perfusion, including: Lactate level > 2 mmol/l, capillary refill time > 3 s or a mottling score ≥ 1.

Exclusion criteria included the impossibility to obtain an adequate hepatic ultrasound assessment; pregnancy; S-wave reversal in hepatic vein Doppler; confirmed or suspected abdominal hypertension; chronic kidney disease in hemodialysis [17]; patients with limitations of therapeutic effort; Child C cirrhosis [18]; moderate to severe tricuspid regurgitation; cardiac arrhythmias; spontaneous ventilation; extracorporeal life support; severe respiratory failure (PaO_2_:FiO_2_ ratio of < 100 mm Hg) and/or need of PEEP > 15 cm H20.

### Ultrasound measurements

A baseline ultrasound assessment was performed as part of standard hemodynamic ICU management. This included echocardiography measures such as color Doppler of the tricuspid valve, left ventricular outflow tract–velocity time integral (LVOT–VTI), lateral mitral *E*/*E*’ ratio, tricuspid annular plane systolic excursion (TAPSE), tricuspid annular systolic velocity by tissue Doppler imaging and ejection fraction (EF), which was calculated using Simpson’s biplane or Teicholz method.

The middle hepatic vein (MHV) was identified from lateral or mid-subcostal view according to previously published reports [19, 20]. A phased array transducer was used with cardiac presets to facilitate study measurements. The MHV was interrogated 2–3 cm from its junction to the inferior vena cava. Color flow Doppler was used to identify high flow parallel to the ultrasound beam and then pulsed wave Doppler was obtained. The sample volume used was 2–3 mm, with velocity range of 65–80 mm/s, and the intercept angle between the Doppler beam and the vessels’ long axis was lower than 45°. Normal hepatic vein morphology follows a triphasic pattern, including two forward and one backward related. S-wave represents forward flow during early–mid systole, D-wave represents forward flow during early–mid diastole, and A-wave represents backward flow during late diastole [12]. The maximum velocity of D and S waves, as well as the VTI of D and S waves were recorded. All Doppler findings were obtained during the end-expiratory phase of the patients´ respiratory cycle with concurrent multi-lead ECG tracings. Trained operators performed ultrasound measurements using Mindray M9 (Bio-Medical Electronics Co., Shenzhen, China).

### PLR maneuver

**Fig. 1:**
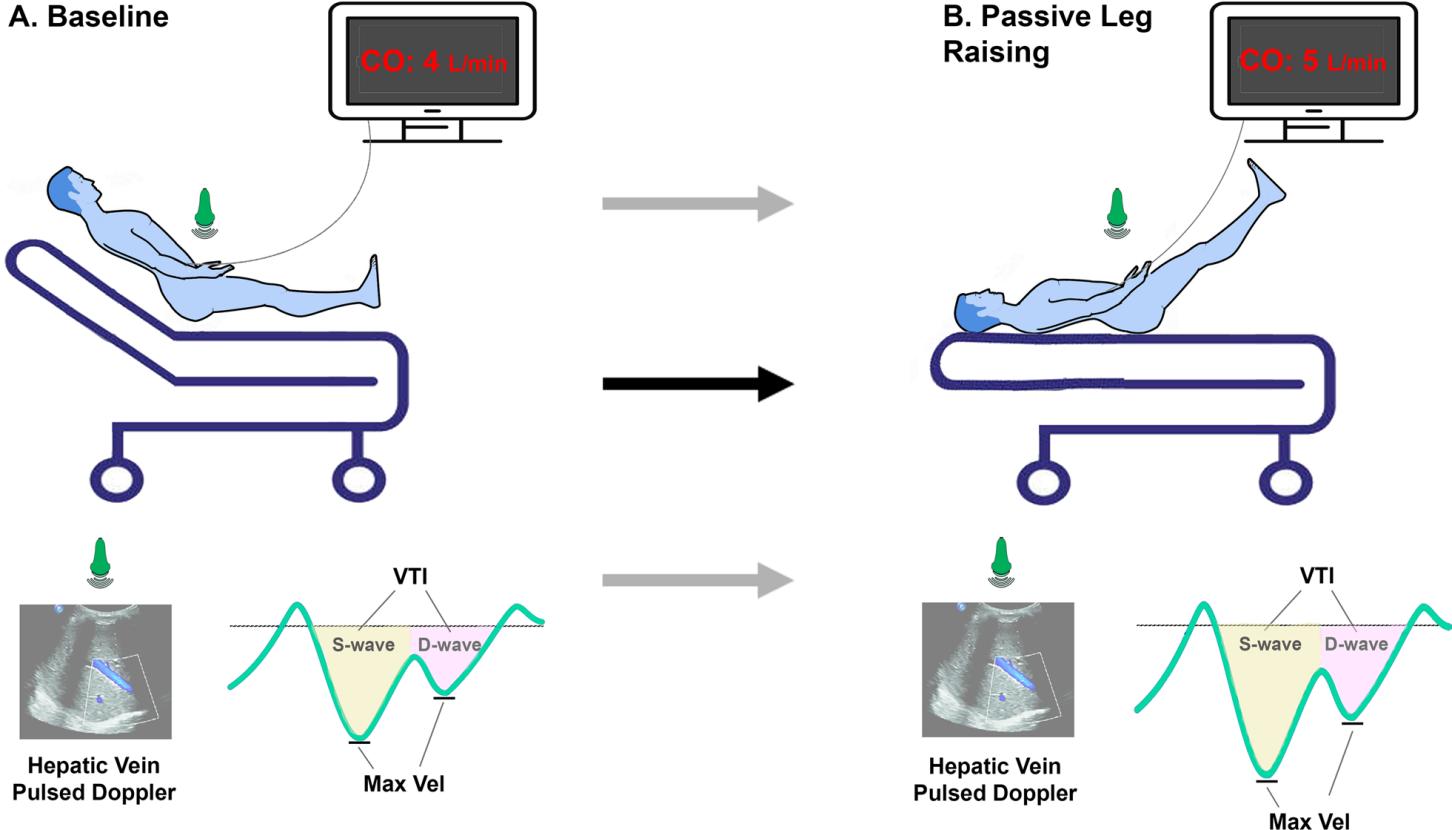
Study protocol. *VTI* velocity time integral

Whenever the attending clinician performed a PLR–CO to assess fluid responsiveness status, the technique was performed according to current recommendations [21]. Baseline patient positioning was in semi-recumbent at 45º angle and ended with the trunk in supine position and both legs elevated [22], as shown in Fig. 1.

Stroke volume and cardiac output were obtained with a continuous pulse contour analysis device (Argos® Cardiac Output Monitor, Retia Medical, USA). The monitors’ refresh rate was set at 20 s to detect meaningful variations during the PLR maneuver. Arterial line transducing adequacy was checked before the PLR maneuver. The HVD measurements were repeated between 1–2 min after leg elevation, at end expiration as well. We did not measure LVOT–VTI after PLR due to the limited timeframe of the maneuver’s effect on CO (1–2 min) [6]. Due to this small window of opportunity, we focused on obtaining proper HVD measurements. During the measurement period, adequacy of arterial line transduction and a minimum tidal volume of 8 ml/kg IBW during controlled mechanical ventilation was ensured. Patients were considered preload responders if CO increased 10% or more after PLR, as suggested by previous studies and recent expert recommendations [23–25].

### Data registry

Demographic data such as age, sex, comorbidities, Acute Physiology and Chronic Health Evaluation II (APACHE II), Sequential Organ Failure Assessment (SOFA) score, Charlson comorbidity index, diagnostic group on admission, laboratory tests, mechanical ventilation parameters [23] and clinically relevant outcomes such as ICU length of stay and 28-day mortality were registered.

### Statistical analysis

Demographic variables are presented as frequency (percentage) and mean ± standard error (SD) or median [interquartile range] as appropriate, depending on data normality distribution. Receiver operating characteristic (ROC) were constructed to assess the diagnostic accuracy of HVD S and D-wave velocity and VTI changes to predict a preload responsive status. Data are presented as the area under the ROC curve (AUC–ROC) ± SD (with a 95% confidence interval), sensitivity (95% CI) and specificity (95% CI). The Youden index (sensitivity + specificity – 1) was calculated to determine the best cutoff values. The grey zone approach was performed to define cases under 90% of sensitivity and specificity (diagnostic tolerance of 10%). AUC–ROC between variables were compared according to Hanley and McNeil method [26]. We performed two exploratory sensitivity analyses: first, using a 15% increase of CO cutoff after PLR, and second, using a 10% cutoff on stroke volume (SV).

Considering previous reports [25, 27], we estimated the sample size comparing an expected AUC–ROC of 0.78 curve with the null hypothesis of 0.50, assuming a two-sided α error of 0.05 and power of 80%. In total, at least 36 cases were required to be included. A *p* value < 0.05 was considered statistically significant. Data were analyzed with Graphpad Prism 10.0 (Graphpad Softwares, La Joya, CA) statistical package.

## Results

From August to December 2023, we screened 50 patients for eligibility and 37 met inclusion and exclusion criteria, as shown in the study flow (Additional File 1). The main patient characteristics at baseline are presented in Table 1. Median age was of 59 ± 15 years, and most had sepsis-related circulatory dysfunction. Median SOFA score was 8 [6–10], and median norepinephrine dose at measurement time was of 0.15 [0.06–0.37] mcg/kg/min. 8% (3/37) of patients were on vasopressin infusion as a second vasopressor. Regarding the tissue perfusion triggers that prompted clinicians to perform a PLR assessment, 40% of patients had abnormal lactate, 46% altered CRT, and 14% an altered mottling score [28].

**Table 1:** Baseline characteristics of study population

Variable	Value
Age (years)	59 ± 15
Male (%)	54%
Diagnosis (%)	
Sepsis/septic shock	81%
Non-cardiac surgery	8%
Other	11%
SOFA score	8 [6–10]
APACHE 2 score	15 [8–22]
Charlson comorbidity index	2 [0–5]
Lactate (mmol/L)	1.5 [1–2.9]
CRT (secs)	3 [2–7]
ScvO2 (%)	83 [69–80]
Delta pCO2 a-v (mmHg)	7 [5–9]
Norepinephrine dose (mcg/kg/min)	0.15 [0.06–0.37]
CVP (mmHg)	10 [7–13]
Use of Vasopressin (%)	8%
ICU LOS (Days)	8 [4–13]
28-day mortality (%)	32%

*CVP*: Central venous pressure, *SOFA*: Sequential Organ Failure Assessment, *ICU LOS*: Intensive care unit length of stay, *CRF* Capillary refill time, *ScvO2* Central venous oxygen saturation, *APACHE II* Acute Physiology and Chronic Health Evaluation II

62% of the patients were preload responsive defined by a 10% increase in CO after PLR. Table 2 shows key hemodynamic, echocardiographic and HV Doppler variables in preload responsive and unresponsive patients. In 11% (4/37) of patients, LVOT–VTI was not assessable due to poor 5-chamber view window. Additional File 2 shows key ventilatory settings in both study groups, while Additional File 3 shows the relationship between TAPSE and Delta S-wave velocity in the whole study cohort.

**Table 2:** Hemodynamic parameters and ultrasound measurements according to preload responsiveness status

	PR +	PR −	*P* value
Number of patients	62% (23/37)	38% (14/37)	
HR (bpm)	84 ± 21	77 ± 18	0.3
MAP (mmHg)	72 [65–77]	79 [72–88]	0.04
CVP (mmHg)	9 [8–15]	10 [7–15]	0.8
CRT (s)	4 [2–7]	3 [2–5]	0.17
NE dose (mcg/kg/min)	0.1 [0.03–0.23]	0.28 [0.08–0.56]	0.1
Stroke volume (mL)	52.5 ± 21.4	61.1 ± 15.5	0.17
Cardiac output (L/min)	4.3 ± 1.5	5.0 ± 2.1	0.25
LVOT–VTI	16.1 ± 4.3	15.1 ± 4.2	0.51
TAPSE (mm)	19.9 ± 4.1	17.2 ± 3.2	0.045
LVEF (%)	49 ± 13	51 ± 11	0.59
*E*/*E*’ ratio	7.7 ± 2.5	8.3 ± 2.5	0.5
Baseline S-wave velocity (m/s)	19 [16–23.1]	21 [17–24.7]	0.34
Baseline D-wave velocity (m/s)	16.5 [13.6–17.7]	17.1 [13–20.4]	0.58
Baseline S-wave VTI (cm)	4.1 [3.5–4.9]	4.0 [3.4–5.8]	0.88
Baseline D-wave VTI (cm)	3.1 [1.9–4.6]	3.5 [2.1–4.9]	0.68

*HR* heart rate, *MAP* mean arterial pressure, *CVP* central venous pressure, *CRT* capillary refill time, *NE* norepinephrine, *LVOT–VTI* left ventricular outflow tract–velocity time integral, *TAPSE* tricuspid annular plane systolic excursion, *LVEF* Left ventricle ejection fraction, *E/E’ ratio* mitral peak Doppler E-wave to peak mitral annulus velocity ratio, *VTI* velocity time integral, *PR* preload responsiveness status

**Fig. 2:**
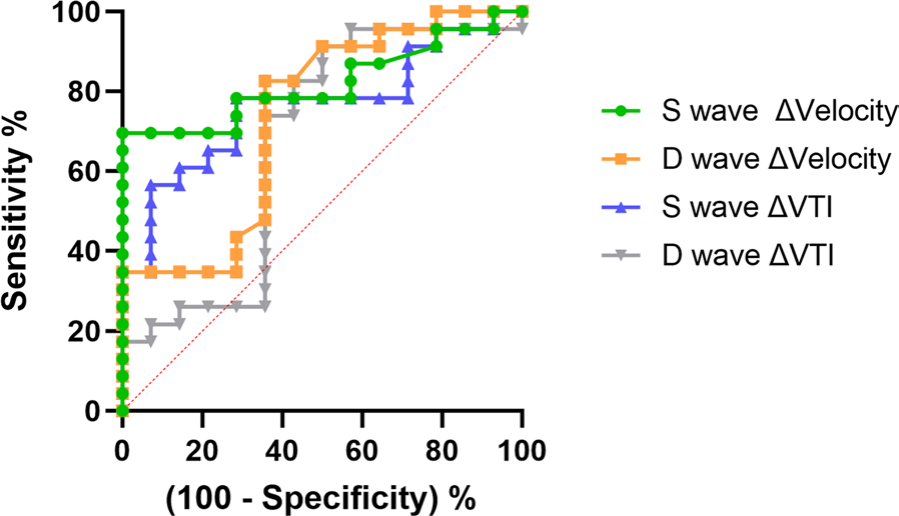
Receiver operating curves of different hepatic vein Doppler parameters to identify preload responsiveness (defined by an increase of CO > 10%). *VTI* velocity time integral

Figure 2 shows ROC curves of the different HVD velocities studied to predict preload responsiveness status. Among them, Delta of S-wave velocity had the higher AUC–ROC (0.82 ± 0.07, *p* = 0.001), as shown in Table 3, followed by delta S-wave VTI (0.76 ± 0.08, *p* = 0.008). No statistically significant differences were found when comparing AUC–ROC of the S-waves related variables (*p* = 0.58). The other variables studied (delta D-wave velocity and delta VTI D-wave), had lower diagnostic yield. Additional File 4 and 5 show ROC curves, AUC–ROC and optimal cutoff values to predict an increase of > 10% of stroke volume of the studied parameters. Additional Files 6 and 7 show ROC curves, AUC–ROC and optimal cutoff values to predict an increase of > 15% of CO, showing similar diagnostic accuracies for Delta S-wave velocity in these contexts.

**Table 3:** Diagnostic accuracy of preload responsiveness (defined by an increase of CO > 10%), and selected cutoff values for hepatic vein Doppler measurements

	AUC–ROC	95% CI	*p* value	Best Cutoff (%)	Sensitivity (%)	Specificity (%)	LR +	LR−
Delta S-wave velocity	0.82 ± 0.07	0.68–0.96	0.001	20	69.6 (49.1–84.4)	92.8 (68.5–99.6)	9.7	0.33
Delta D-wave velocity	0.73 ± 0.09	0.56–0.91	0.02	2.1	82.6 (62.8–93)	64.3 (38.8–83.7	2.3	0.27
Delta S-wave VTI	0.76 ± 0.08	0.61–0.92	0.008	> 25	56.5 (36.8–74.4)	92.8 (68.5–99.6)	7.9	0.47
Delta D-wave VTI	0.67 ± 0.1	0.47–0.87	0.09	> 2.1	73.9 (53.5–87-5)	64.3 (38.8–83.6)	2.1	0.4

*AUC–ROC* area under curve–receiver operator characteristic, *LR* likelihood ratio, *VTI* velocity time integral

**Fig. 3:**
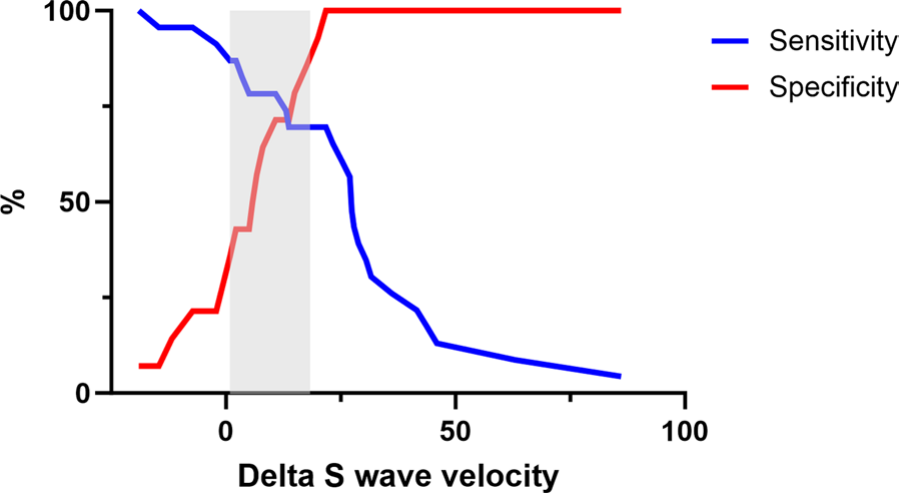
Grey-zone approach of delta-velocity of S-wave hepatic vein Doppler

The optimal cutoff value was obtained for each variable through the Youden index. A 20% increase in the maximum S wave velocity after PLR showed a sensitivity of 69.6 (49.1–84.4) and specificity of 92.8 (68.5–99.6) to detect preload responsiveness, as shown in Table 3. Figure 3 shows the grey-zone approach for delta S-wave velocity, in which 33% of the study group was located between the grey-zone limits. Additional File 8 shows a plot of individual values of different HVD parameters vs. delta CO, which shows the best correlation with Delta S-wave velocity.

## Discussion

The main results of our study can be summarized as follows. A change of 20% of S-wave peak velocity or 25% of S-wave VTI may identify preload responsiveness during a PLR maneuver in sedated and mechanically ventilated critically ill patients. Analysis of D-wave velocities had worse predictive capacity.

The hemodynamic effects of a PLR maneuver have been estimated to mimic a fluid challenge of approximately 300 ml [21]. Current expert recommendations suggest performing the test from a semi-recumbent rather than from supine position, to induce shifts from two vascular beds (the legs and the splanchnic region) and enhance diagnostic accuracy [21]. This was hypothesized in the study by Jabot et al., in which sequential postural changes from semirecumbent to supine and from supine to leg elevation had similar net effect to that of the entire maneuver performed at once [22]. The authors inferred that the scaffolded approach isolated the relative contributions of both vascular territories (splanchnic and legs), but unfortunately, it wasn’t measured [22]. The current study provides direct assessment on the impact of PLR on splanchnic flow through Doppler assessment, potentially confirming this hypothesis. In this sense, the fact that significant changes in flow-related measurements of the splanchnic region accurately predicted changes in CO, support that the contribution of this venous reservoir is relevant, at least in this clinical context. Future research endeavors should further investigate the relative contribution of each territory in this and other clinical contexts.

Contemporary research has assessed the accuracy of splanchnic Doppler signals to predict preload responsiveness. Cheong et al. analyzed the utility of portal vein pulsatility index (PVPi), finding that a value of PVPi > 32% predicted fluid unresponsiveness with a sensitivity 30.8% (17–47.6%) and specificity of 100% (85.8–100) [29]. On the other hand, Wu et al. analyzed absolute values of S and D waves of HVD, finding higher delta S-wave velocity on patients where cardiac output increased after volume expansion as compared to those where it did not (30.1 cm/s ± 10.2 to 37.1 cm/s ± 12.5; *p* < 0.003) [30], moreover, no diagnostic accuracy was analyzed. Our study further expands the results obtained by these previous researchers, since they aim specifically at interrogating Doppler waves that represent forward flow to the atrium and are near inferior vena cava drainage, as compared to portal vein. Thus, we believe it has a stronger physiological background and could interrogate more directly changes in VR, explaining their increased accuracy.

Multiple tests to predict preload responsiveness have focused on measuring arterial flow-related variables to identify markers of CO increase [31], such as pulse pressure or stroke volume. Only two proposed preload responsiveness tests have focused on interrogating the venous side of circulation, namely SVC and IVC diameter variability [32, 33]. However, both present technical and conceptual challenges. The former requires transesophageal ultrasound (US) measurements [34], hindering its’ widespread applicability, while the latter has multiple technical drawbacks related to insonation angle, IVC sphericity and venous compliance, limiting its’ diagnostic accuracy [35]. The novel approach proposed on this study, which focuses on blood flow variations measured through pulsed Doppler rather than anatomical variations, such as diameter, could help overcome these issues, and approach indirectly to VR changes. Due to the angulation required, obtaining flow patterns from the IVC is not feasible, but the HV provides an adequate US assessment site that drains directly into the IVC without major valves or obstructions, becoming a valuable and emerging window for hemodynamic assessment.

Recently, the visual inspection of HVD has also been studied as a marker for venous congestion or fluid intolerance, being a pivotal component of the VExUS score [36]. S-wave reversal, which represents retrograde flow, has been proposed as a marker of hepatosplanchnic congestion. Altered VexUS has correlated to key hemodynamic parameters such as high CVP values [37], and could predict the risk of acute kidney injury in different contexts. HVD has also been used as a marker of venous congestion in heart failure context with prognostic implications as well [38]. As proposed by Kenny et al., the dynamic assessment of waveform patterns (i.e., increase in D wave or reversion of S wave) through a PLR maneuver or fluid challenge could aid in identifying potential congestion [39, 40]. Thus, HVD could be a fairly unique tool able to assess not only fluid responsiveness as seen this manuscript, but also assess fluid tolerance, which is of growing clinical importance [41, 42]. Moreover, future research efforts should answer these areas of uncertainty.

Of note, in this cohort, preload responsive patients had significantly higher baseline TAPSE readings as compared to non-responders (although median values were within normal range). Thus, it could be argued that the difference in preload responsiveness status was determined because patients had better right ventricular function, determining a steeper cardiac function curve. Recent reports such as Zhang et al. have shown that TAPSE correlates with HVD-derived indexes such as the systolic filling fraction [S-wave velocity/(S-wave + D-wave velocities)] [20]. Our results show that delta S-wave velocity correlates with TAPSE (Additional File 3). Both phenomena could be explained by the fact that during ventricular systole the tricuspid annulus moves inward to the cardiac apex, which causes a further anterograde flow during systole (thus reflected in the S wave of HVD) [14]. Moreover, this study was not designed to address this question, and clinical interpretation should be made with caution considering other key echocardiographic results obtained, but certainly it deserves further exploration.

This study has several strengths. First, it’s bicentric nature. Second, delta S-wave velocity presented an adequate AUC–ROC (> 0.8), with less than 40% of measurements in the gray zone of diagnostic tolerance. This becomes particularly relevant when considering the easiness of image acquisition, as compared to LVOT–VTI. In fact, Spiegel et al. only reported 7.9% of inadequate HVD window in a cohort of patients admitted to ICU [43], similar rate to that shown by Prager et al. in a cohort of septic shock patients [15]. Even though our study was not designed to assess image acquisition feasibility of HVD, we excluded only 4% of patients for this reason out of the eligible patients screened (Additional File 1).This contrasts significantly to the prevalence of inadequate windows to obtain LVOT–VTI, which can amount up to 22% [44, 45]. Added to the non-invasive nature of the technique, no requirement of CO monitor and its’ easier learning curve, HVD could emerge as a valuable tool at the bedside assessment of critically ill patients.

This study has several limitations. First, as all ultrasound-based assessments, the technique is operator dependent and intra or inter-observer variability could occur. Second, we decided to assess changes in CO through a PLR rather than the more classic design of administrating a fluid challenge [25] to assess fluid responsiveness. Even though the fluid challenge has been praised as a gold-standard technique, both the physiological determinants of PLR (± 300 ml of autotransfusion) and the diagnostic accuracy of its’ derived measurements (sensitivities and specificities > 85%) [24], provide a comparable alternative. This approach has already been used in the contemporary literature of diagnostic accuracy of preload responsiveness tests [46, 47], and has the added benefit of avoiding potential deleterious fluids [48, 49] to patients which are preload unresponsive. Another potential criticism could be the use of non-calibrated CO monitors such as those used in this study. Moreover, the technical determinants (i.e., pulse contour analysis) for tracking relative changes of CO are the same as those used in transpulmonary thermodilution (TPTD), and the least significant changes of CO variability has been estimated around 1%, significantly under our detection threshold of PR of 10% [50]. The multibeat pulse contour monitoring used has a short refresh rate (20 s), and presents an adequate ability to capture quick and relative changes such as those happening during a PLR, as shown in recent studies [51, 52]. There was a predominance of septic patients, which could present with higher rates of preload responsiveness [53]. Thus, this could preclude extrapolation to other diagnostic groups of circulatory failure. Since our study was the first assessment of the diagnostic accuracy of this novel technique, we decided to avoid potential physiological confounders, such as right sided valvular diseases, light sedation or patients with respiratory efforts, which could introduce bias or error and preclude a correct interpretation of results.

Future studies should confirm these results and test the diagnostic capacity of HVD in broader contexts of critical illness, such as patients with spontaneous breathing, light sedation, arrythmias or valvular diseases. Aswell, the usefulness of the dynamic assessment of fluid intolerance through identification of flow patterns of S and D waves in concomitance with preload responsiveness could aid clinicians on identifying the best balance between risk/benefit ratio on fluid administration. Finally, future studies could compare, through hand-tracking devices, the difference on number of movements and distance travelled between HVD and LVOT–VTI during a PLR maneuver [54].

In conclusion, in sedated and mechanically ventilated critically ill patients, dynamic changes of S-wave on HVD after a PLR maneuver had a suitable predictable capacity to identify preload responsiveness. This technique could become valuable in scenarios of basic hemodynamic monitoring and when cardiac US is not feasible. Future studies should confirm these results and their relationship with dynamic venous congestion assessment.

### Supplementary Information

Additional File 1. Study flow

Additional File 2. Ventilatory parameters of the study cohort.

Additional File 3. Linear relationship between Delta S-wave velocity and TAPSE.

Additional File 4. Diagnostic accuracy of preload responsiveness (defined by an increase of stroke volume > 10%), and selected cutoff values for hepatic vein Doppler measurements.

Additional File 5. Receiver operating curves of different hepatic vein Doppler parameters to identify preload responsiveness (defined by an increase of stroke volume > 10%).

Additional File 6. Diagnostic accuracy of preload responsiveness (defined by an increase of cardiac output > 15%), and selected cutoff values for hepatic vein Doppler measurements

Additional File 7. Receiver operating curves of different hepatic vein Doppler parameters to identify preload responsiveness (defined by an increase of cardiac output  > 15%).

Additional File 8. Linear relationship between HVD velocities variation and cardiac output variation. CO: cardiac output; VTI: velocity time integral.

## Data Availability

The data sets are available from the corresponding author on reasonable request.

## Abbreviations

CO: Cardiac output
PLR: Passive leg raising
ICU: Intensive care unit
LVOT–VTI: Left ventricular outflow tract–velocity time integral
VR: Venous return
HVD: Hepatic vein Doppler
PLR–CO: PLR with cardiac output assessment
MAP: Mean arterial pressure
PEEP: Positive end-expiratory pressure
TAPSE: Tricuspid annular plane systolic excursion
EF: Ejection fraction
MHV: Middle hepatic vein
VTI: Velocity time integral
IBW: Ideal body weight
APACHE-II: Acute physiology and chronic health disease classification system II
SOFA: Sequential organ failure assessment score
AUC–ROC: Area under the curve receiver‐operating characteristic
ROC: Receiver‐operating characteristic
PVPi: Portal vein pulsatility index
SVC: Superior vena cava
IVC: Inferior vena cava
US: Ultrasound
VexUS: Venous excess ultrasound score
TPTD: Transpulmonary thermodilution
