# Supplementary figures and images for: Dynamic changes of hepatic vein Doppler velocities predict preload responsiveness in mechanically ventilated critically ill patients
Source: Intensive Care Med Exp. 2024 May 8;12:46. doi: 10.1186/s40635-024-00631-w (PMC11078902; doi:10.1186/s40635-024-00631-w)

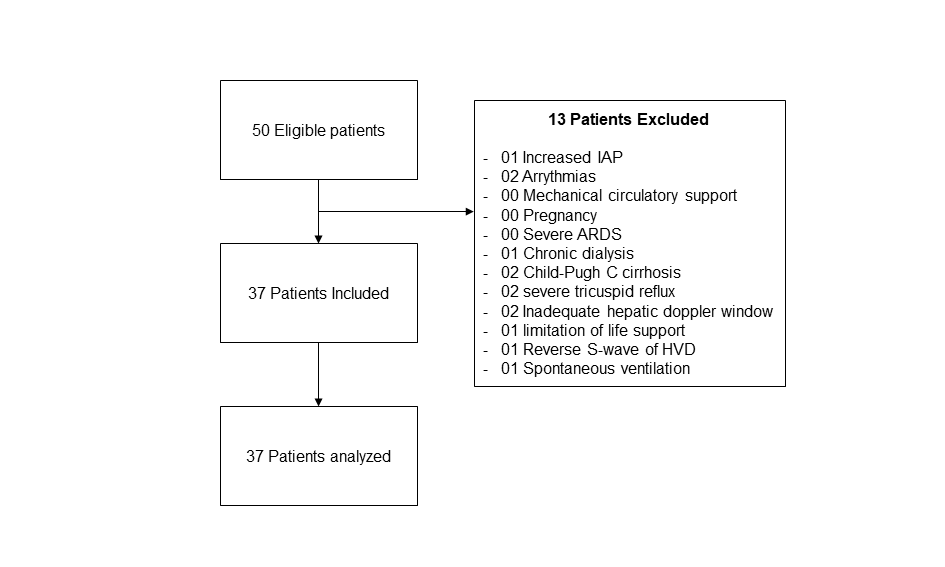

Supplement: Supplementary file 1 — Additional File 1. Study flow [file 40635_2024_631_MOESM1_ESM.tif]

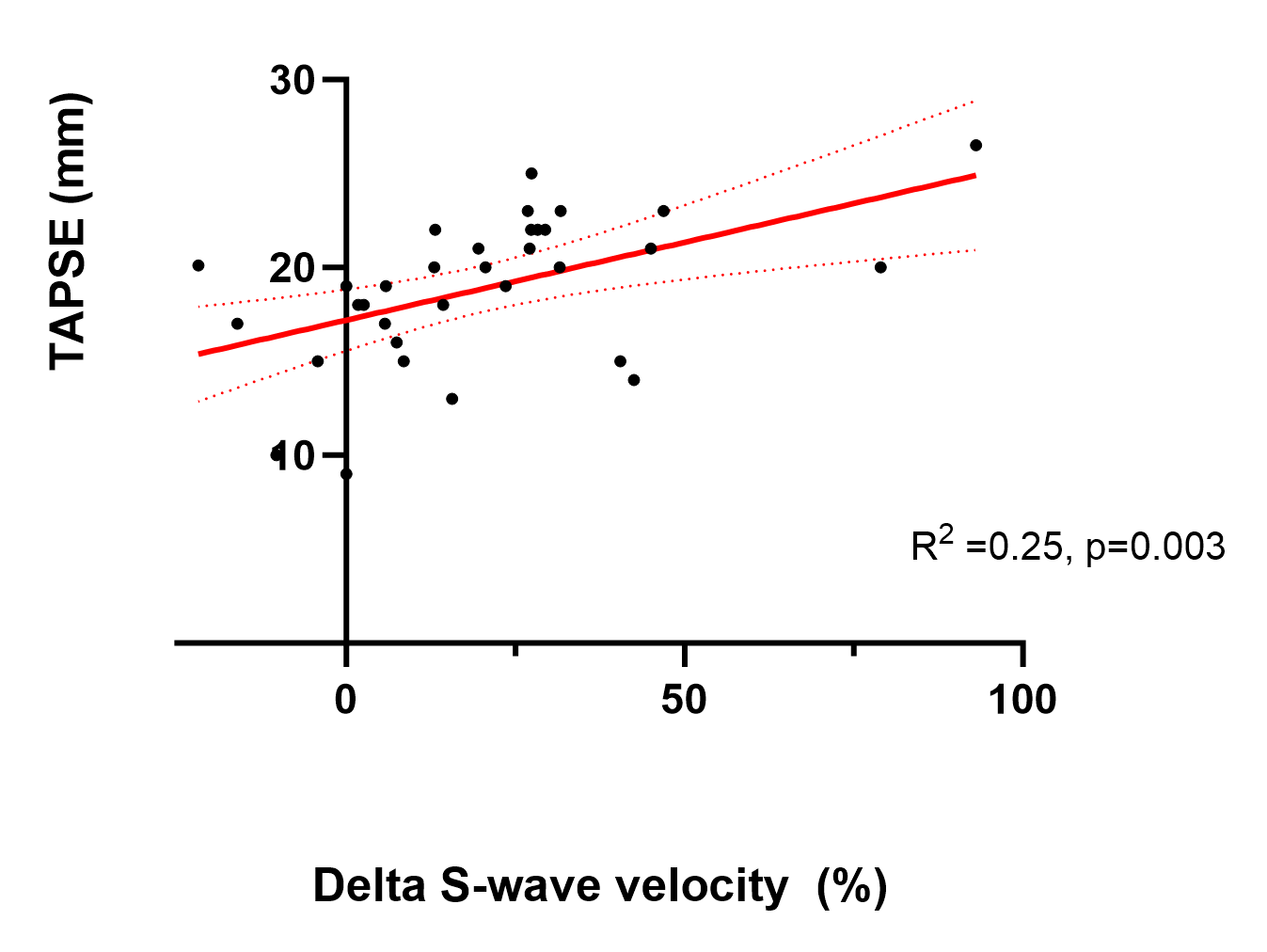

Supplement: Supplementary file 3 — Additional File 3. Linear relationship between Delta S-wave velocity and TAPSE. [file 40635_2024_631_MOESM3_ESM.tif]

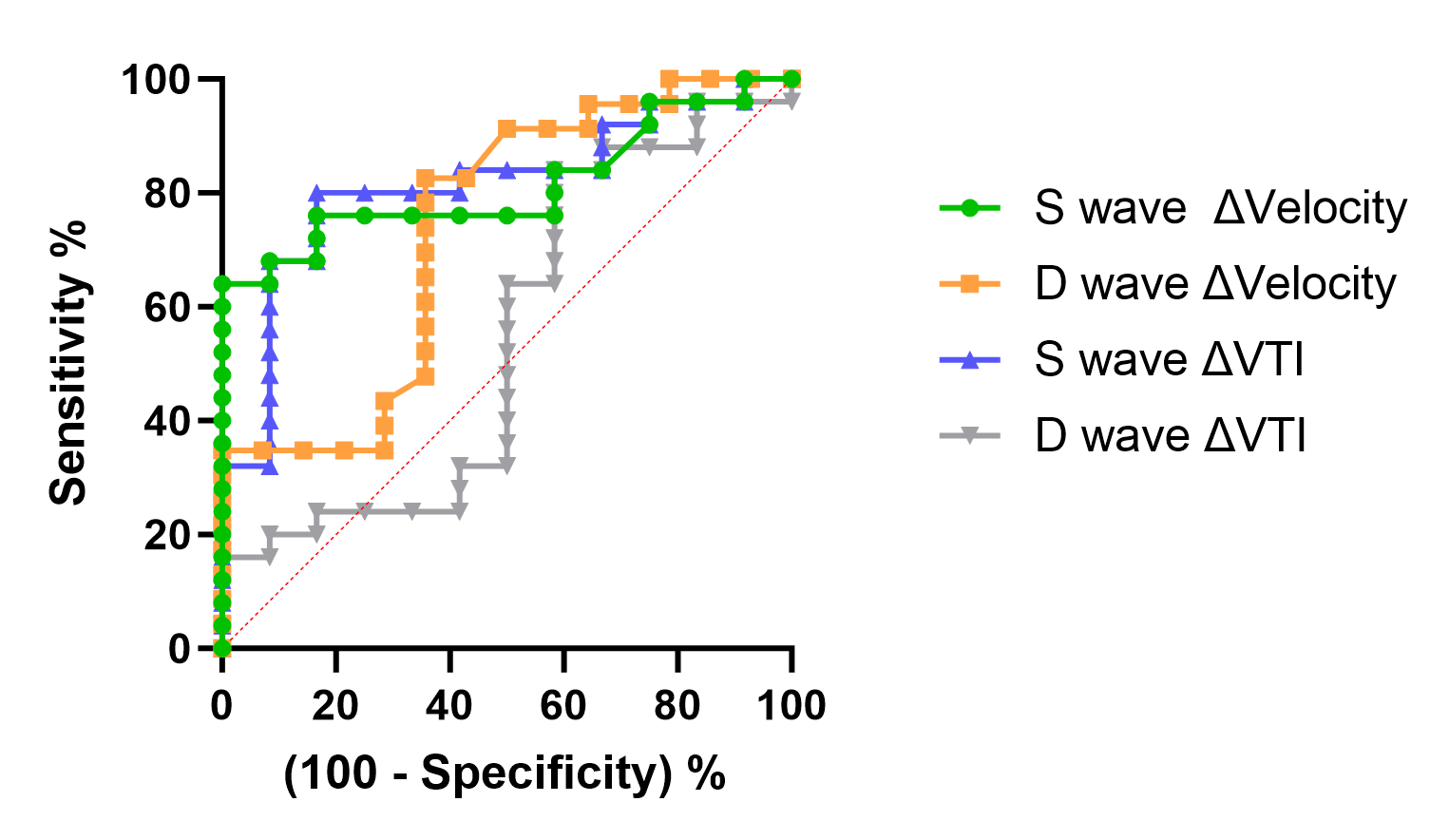

Supplement: Supplementary file 5 — Additional File 5. Receiver operating curves of different hepatic vein Doppler parameters to identify preload responsiveness (defined by an increase of stroke volume > 10%). [file 40635_2024_631_MOESM5_ESM.tif]

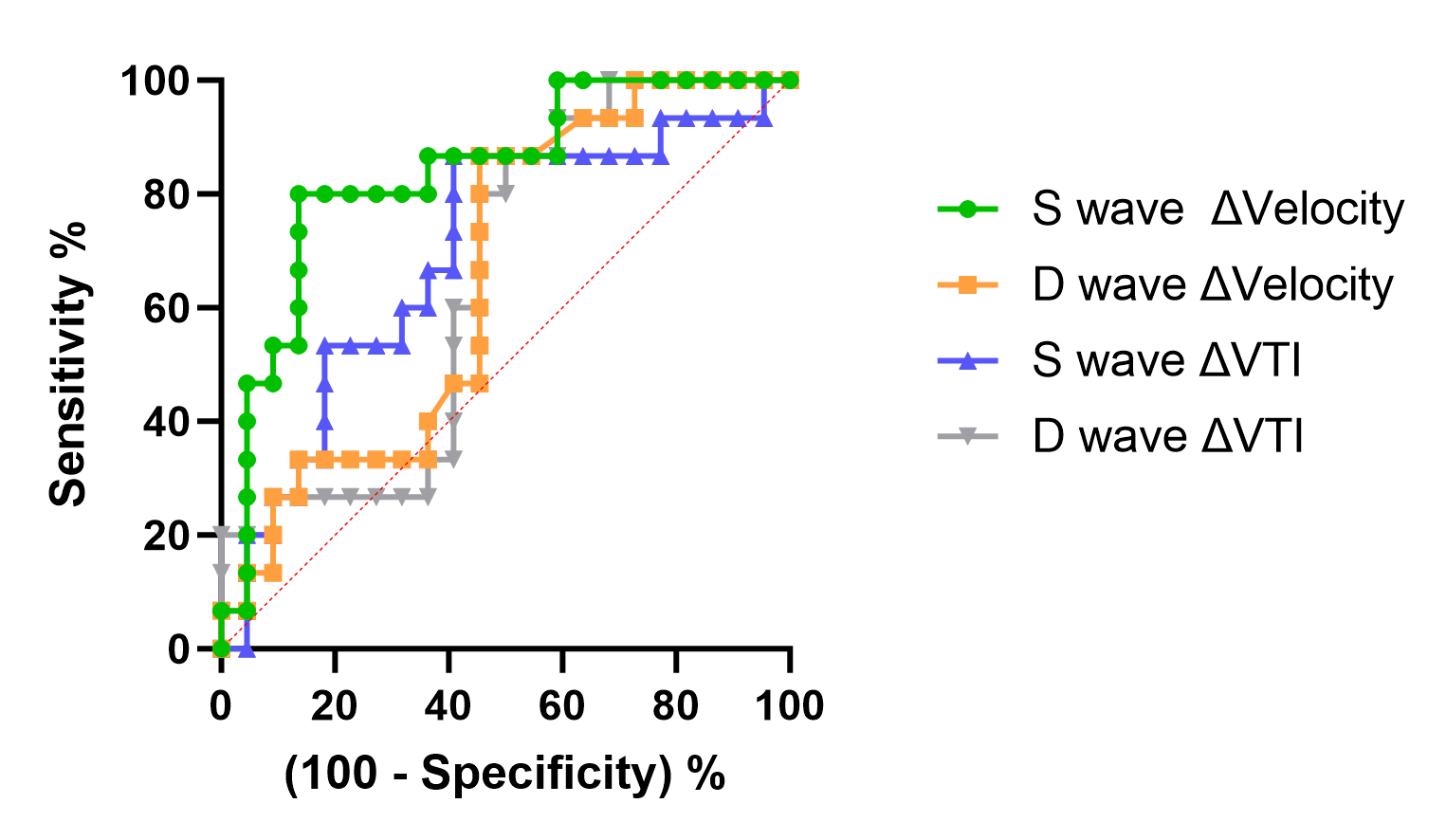

Supplement: Supplementary file 7 — Additional File 7. Receiver operating curves of different hepatic vein Doppler parameters to identify preload responsiveness (defined by an increase of cardiac output > 15%). [file 40635_2024_631_MOESM7_ESM.tif]

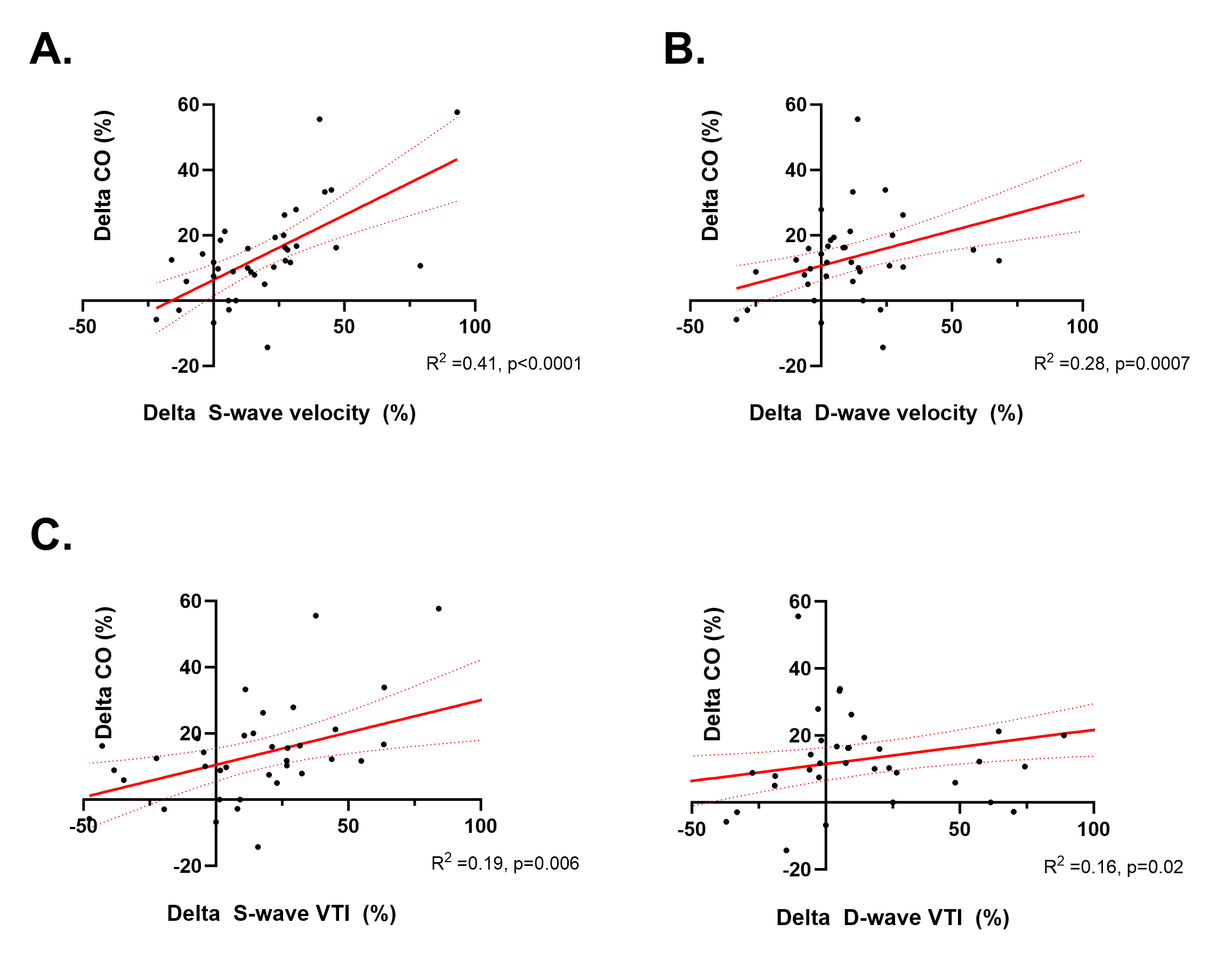

Supplement: Supplementary file 8 — Additional File 8. Linear relationship between HVD velocities variation and cardiac output variation. CO: cardiac output; VTI: velocity time integral. [file 40635_2024_631_MOESM8_ESM.tif]
