# Supplementary material for: Dynamic changes of hepatic vein Doppler velocities predict preload responsiveness in mechanically ventilated critically ill patients
Source: Intensive Care Med Exp. 2024 May 8;12:46. doi: 10.1186/s40635-024-00631-w (PMC11078902; doi:10.1186/s40635-024-00631-w)
Supplement: Supplementary file 2 — Additional File 2. Ventilatory parameters of the study cohort. [file 40635_2024_631_MOESM2_ESM.docx]

Additional File 2: Ventilatory parameters of the study cohort, according to preload responsiveness status.

| Variable | PR+ | PR- | p-value |
| --- | --- | --- | --- |
| Tidal Volume (mL) | 450 [380-500] | 420 [375-490] | 0.4 |
| Plateau Pressure (cmH20) | 18 [15-21] | 18 [16-21] | 0.8 |
| PEEP (cmH20) | 6 [6-7] | 7 [6-8] | 0.14 |
| PaO2:FiO2 ratio | 306 [215-274] | 270 [185-390] | 0.8 |
| Respiratory Rate (rpm) | 22 [20-24] | 23 [20-26] | 0.5 |
| Heart Rate/Respiratory Rate | 3.5 [2.6-4] | 3.8 [3.2-4.1] | 0.5 |
| Controlled Mechanical Ventilation (%) | 100% | 100% | 1.0 |
| SAS 2 or less (%) | 100% | 100% | 1.0 |

PR: preload responder; PEEP: positive end expiratory pressure; SAS: sedation-agitation scale.
