# Supplementary material for: Dynamic changes of hepatic vein Doppler velocities predict preload responsiveness in mechanically ventilated critically ill patients
Source: Intensive Care Med Exp. 2024 May 8;12:46. doi: 10.1186/s40635-024-00631-w (PMC11078902; doi:10.1186/s40635-024-00631-w)
Supplement: Supplementary file 4 — Additional File 4. Diagnostic accuracy of preload responsiveness (defined by an increase of stroke volume > 10%), and selected cutoff values for hepatic vein Doppler measurements. [file 40635_2024_631_MOESM4_ESM.docx]

**Additional File 1: Diagnostic accuracy of preload responsiveness (defined by an increase of SV >10%), and selected cut-off values for hepatic vein doppler measurements**

|  | AUC ROC | 95% CI | p-value | Best Cut-off | Sensitivity (%) | Specificity (%) | LR+ | LR- |
| --- | --- | --- | --- | --- | --- | --- | --- | --- |
| Delta S-wave velocity | 0.81 ± 0.07 | 0.67-0.94 | 0.002 | 20 | 64.0 (44.5-  79.8) | 91.7 (64.5-99.6) | 7.71 | 0.39 |
| Delta D-wave velocity | 0.73 ± 0.09 | 0.56-0.91 | 0.018 | 2.08 | 82.6 (62.8-93) | 64.3 (38.8-83.7 | 2.3 | 0.27 |
| Delta S-wave VTI | 0.81 ± 0.07 | 0.67-0.96 | 0.002 | 16.8 | 68 (48.14-82.8) | 91.7 (64.6-99.6) | 8.2 | 0.35 |
| Delta D-wave VTI | 0.54 ± 0.11 | 0.33-0.76 | 0.65 | 2.07 | 64 (44.5-79.8) | 50 (25.4-74.6) | 1.28 | 0.72 |

SV: stroke volume; AUCROC: area under curve receiver operator characteristic, LR: likelihood ratio; VTI: Velocity time integral.
