# Supplementary material for: Dynamic changes of hepatic vein Doppler velocities predict preload responsiveness in mechanically ventilated critically ill patients
Source: Intensive Care Med Exp. 2024 May 8;12:46. doi: 10.1186/s40635-024-00631-w (PMC11078902; doi:10.1186/s40635-024-00631-w)
Supplement: Supplementary file 6 — Additional File 6. Diagnostic accuracy of preload responsiveness (defined by an increase of cardiac output > 15%), and selected cutoff values for hepatic vein Doppler measurements [file 40635_2024_631_MOESM6_ESM.docx]

**Additional File 6: Diagnostic accuracy of preload responsiveness (defined by an increase of cardiac output >15%), and selected cut-off values for hepatic vein doppler measurements**

|  | AUC ROC | 95% CI | p-value | Best Cut-off (%) | Sensitivity (%) | Specificity (%) | LR+ | LR- |
| --- | --- | --- | --- | --- | --- | --- | --- | --- |
| Delta S-wave velocity | 0.84 ± 0.07 | 0.70-0.97 | 0.0002 | 24 | 80 (54.8-  93) | 86 (66.7-95.3) | 5.9 | 0.23 |
| Delta D-wave velocity | 0.66 ± 0.09 | 0.48-0.83 | 0.11 | 2.4 | 86.7 (62-97) | 55 (35-73) | 1.9 | 0.24 |
| Delta S-wave VTI | 0.80 ± 0.09 | 0.52-0.87 | 0.044 | 10 | 87 (62-98) | 59 (338.7-76.7) | 2.1 | 0.23 |
| Delta D-wave VTI | 0.65 ± 0.09 | 0.47-0.83 | 0.12 | 2.07 | 80 (55-93) | 55 (35-73) | 1.76 | 0.37 |

CO: cardiac output; AUCROC: area under curve receiver operator characteristic, LR: likelihood ratio; VTI: Velocity time integral.
